# Next Generation Therapeutics for the Treatment of Myelofibrosis

**DOI:** 10.3390/cells10051034

**Published:** 2021-04-27

**Authors:** Douglas Tremblay, John Mascarenhas

**Affiliations:** Tisch Cancer Institute, Division of Hematology/Oncology, Icahn School of Medicine at Mount Sinai, One Gustave L Levy Place, Box 1079, New York, NY 10029, USA; douglas.tremblay@mountsinai.org

**Keywords:** myelofibrosis, BET, BCL-2/xL, LSD1, telomerase, TGFb, MDM2, CD123, PI3K, PRM-151, Aurora kinase

## Abstract

Myelofibrosis is a myeloproliferative neoplasm characterized by splenomegaly, constitutional symptoms, bone marrow fibrosis, and a propensity towards transformation to acute leukemia. JAK inhibitors are the only approved therapy for myelofibrosis and have been successful in reducing spleen and symptom burden. However, they do not significantly impact disease progression and many patients are ineligible due to coexisting cytopenias. Patients who are refractory to JAK inhibition also have a dismal survival. Therefore, non-JAK inhibitor-based therapies are being explored in pre-clinical and clinical settings. In this review, we discuss novel treatments in development for myelofibrosis with targets outside of the JAK-STAT pathway. We focus on the mechanism, preclinical rationale, and available clinical efficacy and safety information of relevant agents including those that target apoptosis (navitoclax, KRT-232, LCL-161, imetelstat), epigenetic modulation (CPI-0610, bomedemstat), the bone marrow microenvironment (PRM-151, AVID-200, alisertib), signal transduction pathways (parsaclisib), and miscellaneous agents (tagraxofusp. luspatercept). We also provide commentary on the future of therapeutic development in myelofibrosis.

## 1. Introduction

Myelofibrosis (MF) is a clonal hematological malignancy which is pathologically characterized by bone marrow fibrosis, extramedullary hematopoeisis, and an overactive JAK-STAT pathway and clinically characterized by splenomegaly, cytopenias, and constitutional symptoms including fever, night sweats, and weight loss [1]. These constitutional symptoms can be debilitating, compromising quality of life in MF patients [2]. MF can either rise de novo, termed primary MF (PMF), or from an antecedent MPN such as polycythemia vera (PV) or essential thrombocythemia (ET), termed post-PV/ET MF [3]. MF originates at the level of the multipotent hematopoietic stem cell (HSC). The majority of patients harbor a driver mutation (*JAK2*, *MPL*, or *CALR*) and can also harbor non-driver mutations (e.g., *ASXL1*, *EZH2*, *IDH1/2*, *SRSF2*, *DNMT3A*, *TET2*) [4]. A defective stem cell niche, which includes non-malignant cells within the bone marrow microenvironment, contributes to the development of MF via multiple mechanisms including increased inflammatory cytokine production, abnormal stem cell trafficking, and aberrant myeloid cell proliferation [5]. The only therapy with curative potential in MF is allogeneic hematopoietic stem cell transplantation (HSCT) [6], although this therapy is not available to the majority of patients because of advanced age or comorbidities.

Currently, there are two United States Food and Drug Administration (FDA) approved therapies for MF: ruxolitinib (Jakafi, Incyte, Wilmington, DE, USA) and fedratinib (Inrebic, Bristol-Myers Squibb, New York, NY, USA) [7,8]. Both are JAK inhibitors (JAKi), with ruxolitinib demonstrating equipotent inhibition of both JAK1 and JAK2 while fedratinib demonstrates selectivity inhibition for JAK2 but also FLT3 [9]. In the case of ruxolitinib, approval was based on the pivotal COMFORT I and II trials. These trials demonstrated that ruxolitinib was superior to placebo, in COMFORT I, and best available therapy (BAT), in COMFORT II, in terms of spleen volume reduction (SVR) and symptom improvement [10,11]. Fedratinib has been evaluated in the front-line setting in the JAKARTA study and in patients with ruxolitinib resistance or intolerance and has similarly demonstrated efficacy measured by spleen and symptom improvement [12,13].

Despite the undeniable improvements in patient quality of life with the advent of JAKi, there remains several unmet needs for patients with MF. For one, JAK inhibition leads to on-target myelosuppression, as the erythropoietin and thrombopoietin receptors are JAK-STAT dependent [14]. Clinically, two of the most common treatment emergent adverse events (TEAE) of both ruxolitinib and fedratinib are anemia and thrombocytopenia [10,11,13], which make treatment difficult for patients with disease-related cytopenias. Targeting the JAK-STAT pathway also does not appear to reduce transformation to acute myeloid leukemia, termed MPN blast phase (MPN-BP). In long-term follow up of COMFORT-I and -II, the number of patients who transformed to MPN-BP was not different between those randomized to ruxolitinib or placebo/BAT [15,16]. However, clinical studies are limited as a result of short follow up, especially in the placebo/BAT arm due to crossover design. More compelling evidence that inhibition of the JAK-STAT pathway does not prevent progression to MPN-BP comes from a clonal evolution study of 15 JAKi treated patients where whole-exome sequencing was performed at multiple time points during treatment, allowing for monitoring of clonal evolution over time. In this study, treatment with a JAKi led to an increase in genetic complexity over time, suggesting that JAKi exposure does not significantly prevent clonal evolution and progression to MPN-BP [17]. Because of these limitations, therapeutic development has moved to targets beyond the JAK-STAT pathway in an effort to deplete the MF stem cell and impart a positive impact on the natural history of the disease.

In this review, we describe novel therapies for MF outside of JAKi (Table 1). We will start by briefly describing the current FDA-approved treatment armamentarium for MF, which includes two JAK inhibitors. We also detail the limitations of JAKi therapy for MF. We then outline novel therapies, limiting our focus to agents with clinical trial evidence either published or presented. For each agent, we review their preclinical efficacy, clinical activity, and plans for future development. We then detail urgent unmet needs for patients with MF and describe how novel therapies may address these needs.

## 2. JAK Inhibitors

As previously described, ruxolitinib and fedratinib are the only two approved therapies for MF. National Comprehensive Cancer Network (NCCN) guidelines recommend ruxolitinib for patients with higher risk or symptomatic lower risk disease. Fedratinib is an alternative front-line treatment in higher risk patients with category 2b recommendation. In patients who were treated with ruxolitinib and did not respond or lost a response, fedratinib may also be employed [18]. This management recommendation is informed by the phase 2 open label, single arm JAKARTA-2 study where 97 patients with intermediate or high-risk MF who were either ruxolitinib resistant (67%) or intolerant (33%) were treated with fedratinib 400 mg daily. Of the 83 patients with information available for assessment at 24 weeks, 55% achieved a spleen reduction of at least 35% (SVR_35%_), as measured by imaging. Twenty-nine (53%) of the 55 patients resistant to ruxolitinib and 17 (63%) of 27 patients intolerant to ruxolitinib achieved spleen responses at 24 weeks. The median decrease in spleen volume was 34%. In the 90 evaluable patients for symptom response, 26% achieved a reduction in the MPN Symptom Assessment Form Total Symptom Score of at least 50% (TSS_50%_) [13]. Fedratinib is thus an effective commercially available treatment option in patients with ruxolitinib resistance or intolerance who continue to have symptomatic splenomegaly or constitutional symptoms. On target myelosuppression is associated with fedratinib use, but recent data confirms the safety and efficacy at 400 mg daily in patients with a baseline platelet count of 50–100 × 10^9^/L [19].

A major limitation to JAKi therapy in MF is hematologic TEAE including anemia and thrombocytopenia. However, there are novel JAKi in development which may be useful in these challenging scenarios. Momelotinib is a selective inhibitor of JAK1, JAK2, and ACVR1 which is being developed in anemic MF patients [20]. In preclinical models, inhibition of ACVR1 by momelotinib leads to decreased hepcidin production, which mobilizes sequestered iron and improves erythropoiesis [21]. In the phase III SIMPLIFY trial, which randomized JAKi naïve patients to ruxolitinib or momelotinib, the two treatments were non-inferior for spleen response but not symptom response. However, transfusion requirements and dependence were significantly reduced with momelotinib as compared with ruxolitinib [22]. A phase III trial that evaluated momelotinib in the second line setting after ruxolitinib suboptimal response or TEAE anemia failed to meet its primary endpoint of SVR of at least 35% (SVR_35%_) [23]. Momelotinib is now being evaluated in the phase 3 Momentum study in anemic, symptomatic patients who have previously been treated with a JAKi (NCT04173494). The control arm is danazol and the primary endpoint of this registration study is TSS_50%_ and a key secondary endpoint is anemia response. Pacritinib is a JAK2/IRAK1 inhibitor that is efficacious in MF patients with thrombocytopenia [24]. The phase 3, PERSIST-2 evaluated pacritinib versus BAT, which could include ruxolitinib, in patients with a baseline platelet count of <100 × 10^9^/L. SVR_35%_ was achieved in significantly more patients treated with pacritinib as compared with BAT, particularly in patients with a platelet count of <50 × 10^9^/L [25]. After a full clinical hold due to initial concerns of increased bleeding risk and cardiovascular events compromising overall survival was released, a dose-finding trial was conducted in patients who had failed ruxolitinib in order to identify the lowest effective dose of pacritinib. This phase 2 trial demonstrated that 200 mg BID was the most effective dose resulting in SVR_35%_ rates of 9.3% in this previously JAK inhibitor-treated population. Importantly, in patients with platelets below 50 × 10^9^/L, the SVR_35%_ rate was 16.7% and when considering only evaluable patients in this subgroup, 30.8% achieve an SVR_35%_ [26]. Given promising activity of pacritinib in thrombocytopenic patients, a phase III trial, PACIFICA, is currently accruing MF patients with a platelet count less than 50 × 10^9^/L (NCT03165734) and limited or no prior ruxolitinib exposure and randomizing them to pacritinib 200 mg twice daily versus physician’s choice.

**Figure 1:**
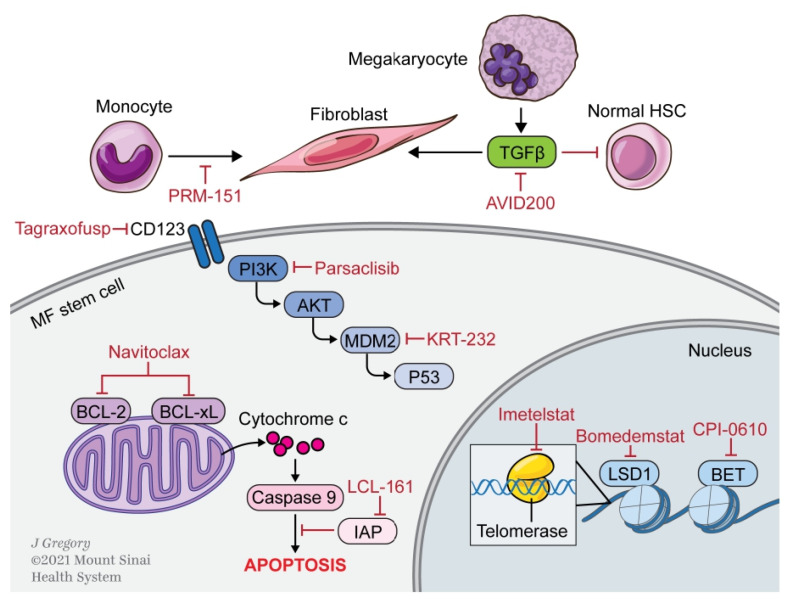
Targets of novel therapeutics in myelofibrosis for agents in clinical development. Multiple pathways are targeted by the next generation of agents for myelofibrosis, including apoptosis, epigenetics, telomerase, and numerous intracellular signaling pathways.

Therapeutic options after ruxolitinib failure are unfortunately limited. As demonstrated in multiple retrospective series, discontinuation of ruxolitinib is associated with a dismal overall survival (OS) of 11–15 months [27,28,29,30]. In addition, some patients do not respond or have an inadequate response to JAKi treatment. Finally, despite advances in symptom and spleen burden with JAKi treatment, progression of disease to MPN-BP is largely unaffected. Therefore, novel therapies are being explored that act on pathways outside the JAK-STAT pathway (Figure 1).

## 3. Novel Therapies

### 3.1. Apoptosis

#### 3.1.1. BCL-2/xL

B-cell lymphoma -2 (BCL-2) family of proteins are anti-apoptotic regulators, inhibiting cytochrome c release from the mitochondria [31]. BCL-xL is a member of the BCL-2 family which is increased in *JAK2-*mutated CD34+ cells from MPN patients compared with healthy controls [32]. In addition, JAK-STAT pathway activation increases BCL-2 and BCL-xL expression [33]. ABT-737 is a BCL-2/BCL-xL inhibitor which has been preclinically evaluated in MPNs. In CD34+ cells from patients with PV, ABT-737 in combination with interferon-α selectively induced apoptosis in *JAK2V617F* mutated cells [34]. In a separate study, ABT-737 in combination with ruxolitinib led to higher apoptotic rate than either individual drug alone [35].

Navitoclax (Abbvie, Chicago, IL, USA) is a BCL-2/BCL-xL inhibitor that has been explored in combination with ruxolitinib in a phase 2 study of 34 patients who had experienced ruxolitinib failure. In terms of safety, all patients experienced a TEAE, with the most common being thrombocytopenia (88%), diarrhea (68%), and fatigue (62%). Thrombocytopenia was manageable with dose modification. At 24 weeks of evaluable patients, 9 patients (27%) achieved a SVR_35%_ and 6 patients (30%) achieved a TSS_50%_. Ten patients (29%) achieved an improvement in bone marrow fibrosis of at least 1 grade at any time during the study. Five patients had a reduction in driver mutation variant allele frequency (VAF) of greater than 20%. Importantly, high molecular risk (HMR) or mutation in 3 or more genes did not impact SVR_35%_, TSS_50%_, or bone marrow improvement [36]. A phase 3, placebo-controlled study comparing the combination of navitoclax and ruxolitinib to ruxolitinib alone in both the JAKi naïve (NCT04472598) and JAKi relapsed/refractory (NCT04468984).

#### 3.1.2. SMAC

Inhibitor of apoptosis proteins (IAPs) control cell survival and are upregulated in numerous cancers. When a cell receives a stimulus to undergo apoptosis, second mitochondria-derived activator of caspases (SMAC) are released in the cytosol and degrade IAPs, which cause pro-apoptotic proteins to be freed, leading to cell death [37]. LCL-161 (Novartis, Basel, Switzerland) is an oral, weekly SMAC mimetic which has been evaluate in a number of malignancies. *JAK2V617F* cells are hypersensitive to LCL-161 in the absence of TNFα. In a *JAK2V617F* murine model, LCL-161 led to reduction in splenomegaly and bone marrow fibrosis. Interestingly, JAK-STAT inhibition in the presence of LCL-161 results in apoptosis in both *JAK2V617F* and wildtype cells, suggesting that combination with a JAKi would detract from selective apoptosis of MF stem cells [38].

LCL-161 has been explored in a phase 2 study of 47 MF patients who were refractory or ineligible to receive a JAKi. The treatment was overall well tolerated with nausea/vomiting, fatigue, and dizziness/vertigo being the most common non-hematologic adverse events (AEs). Grade 3/4 AEs included syncope (n = 2), nausea/vomiting (n = 1), thrombocytopenia (n = 3) and anemia (n = 2). Clinical improvement (CI) in symptoms by IWG-ELN response (Table 2) occurred in 11 patients (23.4%), CI anemia in 6 patients (12.8%), and 1 patient had a CI spleen (2.1%). After a median follow up of 21.1 months, median OS was not reached and the median response duration was 31.5 months [39]. There are no new clinical trials listed on clinicaltrials.gov for LCL-161 in MF at the time of writing this article.

#### 3.1.3. MDM2

Upregulation of the tumor suppressor gene *p53* is an important pathogenic feature of MPNs. In CD34+ cells from MPN patients, treatment with pegylated IFNα-2a causes an increase in apoptosis and phosphorylation/activation of p38 mitogen-activated protein kinase (MAPK), which leads to increase p53 transcription [40]. MDM2 binds p53 and inhibits its activity via multiple mechanisms including ubiquitin mediated proteasome degradation, thereby acting as a negative regulator [41]. Nutlins are MDM2 antagonists which are capable of depleting *JAK2V617F*-mutated MPN stem/progenitor cells in vitro [42,43]. Idasanutlin (RG7388, Roche, Basel, Switzerland) is an oral agent that has been evaluated in a phase 1 trial of 12 PV and ET patients. In this trial, idasanutlin was generally well tolerated although low grade gastrointestinal toxicities were common and led to eventual discontinuation in some patients. The overall response rate (ORR) was 58% and there was rapid reduction in *JAK2V617F* allele burden [44]. This prompted an international phase 2 study of idasanutlin 150 mg daily for 5 days in a 28-day cycles in 27 PV patients who were resistant or intolerant to hydroxyurea. Despite clinical activity in 50% of patients at 32 weeks and the significant reduction in *JAK2V617F* VAF, persistent low-grade gastrointestinal toxicity led to treatment discontinuation in 41% of patients, limiting the future development of this agent in PV [45].

The PV experience with idasanutlin suggests that MDM2 inhibition may be a particularly effective target in other MPNs. KRT-232 (Kartos, Redwood City, CA, USA) is a potent oral MDM2 inhibitor currently in clinical development for the treatment of MF and acute myeloid leukemia (AML). In a phase 2 study of 82 MF patients who are relapsed or refractory to JAKi, KRT-232 was tested in three dosing schedules: 120 mg daily for 21 days on a 28-day schedule, 240 mg daily for 21 days on a 28-day schedule, or 240 mg daily without interruption. Of the 25 evaluable patients at the highest dose level (240 mg daily without interruption), 4 (16%) attained an SVR_35%_ at either week 12 or 24. Only one patient in the second highest dose level achieved an SVR_35%_ and no patients in the lowest dose level had a spleen response. TEAE occurred in 98% of patients, with diarrhea (62%), nausea (38%), vomiting (21%), and abdominal pain (20%) being the most common non-hematologic AEs reported [46]. This clinical trial in relapse/refractory population (NCT03662126) and an additional trial of combination therapy in patients who have a suboptimal JAKi response (NCT04485260) are ongoing.

### 3.2. Epigenetic Modulation

#### 3.2.1. Bromodomain and Extra-Terminal Domain

The BET (bromodomain and extra-terminal domain) family contains four closely related proteins: BRD2, BRD3, BRD4, and BRDt. BET proteins perform diverse regulatory activities affecting RNA polymerase II and ultimately influencing gene expression by recognizing and binding acetylated lysine residues on histone tails. This interaction leads to the localization of BET proteins to discrete chromosomal locations and allows the recruitment of regulatory complexes that influence gene expression [47,48,49]. BRD4 inhibition leads to attenuation of the NF-κB pathway which plays a pivotal role in the pro-inflammatory state that characterizes MF [50]. Pan-BET inhibitors can significantly dampen the inflammatory response of bone marrow-derived macrophages, suppressing expression of interleukin (IL)-6, IFN-b1, IL-1b, IL-12a, CXCL9 and CCL12 [51]. In a preclinical *MPLW515L*-driven murine model of MF, BET inhibition abrogated NF-κB signaling and reduced inflammatory cytokine production. Additionally, combination JAK and BET inhibitor led to synergistic reduction in inflammatory cytokine levels, splenomegaly, and bone marrow fibrosis [52].

CPI-0610 (Constellation Pharmaceuticals, Cambridge, MA) is a potent BET inhibitor in development for MF. It has been explored in the phase 2 MANIFEST study which included 3 arms targeting distinct MF populations: (1) CPI-0610 monotherapy in JAKi refractory/intolerant, (2) CPI-0610 add-on to patients with inadequate response to ruxolitinib, and (3) combination ruxolitinib with CPI-0610 in JAKi naïve patients. In the first arm, 43 patients who are intolerant or refractory to JAKi were given CPI-0610 as monotherapy. Sixteen patients were transfusion dependent at study enrollment. At 24 weeks, median spleen volume change was −17.4%, however no patients had a greater than 35% reduction. One of 12 evaluable patients achieved TSS_50%_. Importantly, 3 out of 14 evaluable patients converted from transfusion dependence to independence. Twenty-seven patients were transfusion independent in a separate cohort. In evaluable patients at week 24, CPI-0610 treatment led SVR_35%_ in 5 out of 21 patients (23.8%) and 9 out of 19 patients (47.4%) achieved TSS_50%_. The most common hematological TEAEs of any grade were thrombocytopenia (25.6%) which was grade 3 in 14% of patients and anemia (11.6%), which was grade 3 in 9.3% of patients [53].

In a separate arm of the MANIFEST study, CPI-0610 was explored as an “add-on” therapy to patients who had a sub-optimal response to ruxolitinib in 44 transfusion-dependent patients and 26 transfusions-independent patients. In the transfusion-dependent cohort, 34.4% of patients converted to transfusion independence. In evaluable patients at week 24, 5 patients (20.8%) had SVR_35%_ and 12 patients (46.2%) had TSS_50%_. In evaluable patients at 24 weeks in the transfusion independent cohort, 4 patients (22.2%) achieved SVR_35%_ and 7 patients (36.8%) achieved TSS_50%_ [54].

Perhaps the most exciting data for CPI-0610 in MF comes from its use in the upfront setting. Eligible patients were JAKi naïve and had a baseline platelet count ≥100 × 10^9^/L. Seventy-eight patients were enrolled at last presented data cut off and were treated with ruxolitinib by platelet count guidelines and CPI-0610. The median SVR was 50% with 67% achieving an SVR_35%_. The benchmark from the COMFORT I study is a SVR_35%_ reached in 42% of patients, for reference [10]. TSS_50%_ was reached in 57% of patients. Importantly, there was at least 1 grade improvement in bone marrow fibrosis in 33% of patients. Anemia and thrombocytopenia were the most common TEAE which occurred in 33% and 32% of patients and was grade 3/4 in 29% and 8%, respectively [55]. The phase 3 MANIFEST-2 study will randomize approximately 310 JAKi naive MF patients to ruxolitinib with either CPI-0610 or placebo (NCT04603495)

#### 3.2.2. LSD1

Lysine-specific demethylase 1 (LSD1) is an epigenetic modifier that regulates gene transcription by removing mono- and dimethyl groups from histone H3. Normal hematopoiesis requires LSD1 and knockdown or pharmacologic inhibition results in expansion of granulomonocytic, erythroid and megakaryocytic progenitors, but attenuates terminal granulopoiesis, erythropoiesis and platelet production. Importantly, this effect is reversible when LSD1 is restored [56]. Bomedemstat (IMG-7289, Imago Biosciences, San Francisco, CA, USA) is an irreversible inhibitor of LSD1. In a *JAK2V617F*-positive MPN mouse model, once daily treatment with bomedemstat improved blood cell counts, reduced spleen volumes, reduced bone marrow fibrosis, and lowered mutant allele burden. Mice also experienced longer survival when treated with bomedemstat. Mechanistically, bomedemstat increased expression of p53 and decreases levels of BCL-xL, resulting in increased apoptosis of *JAK2V617F* cells [57].

Bomedemstat has been evaluated in a phase 1/2 study including MF patients resistant or intolerant to ruxolitinib or fedratinib. Patients must have had a platelet count of at least 100 × 10^9^/L and dose titrations were based on patient platelet count to target 50–75 × 10^9^/L. Forty-nine patients were enrolled at the most recent analysis, with 51% harboring an HMR mutation. Bomedemstat was well tolerated, with no dose limiting toxicities were seen at 6 mg/kg. The most common non-hematologic toxicity was dysgeusia presenting in 35% of patients. Of 32 patients evaluable for symptom response at week 12, 78% had a reduction in TSS with 25% achieving a TSS_50%_. Reduction in spleen volume was noted in 84% of 14 evaluable patients at week 12, with 14% achieving a SVR_35%_. Interesting, there was a decrease in somatic mutations in approximately one-third of patients [58]. This trial is ongoing (NCT03136185).

### 3.3. Microenvironment

#### 3.3.1. Pentraxin-2

Pentraxin-2 (also called serum amyloid P) is an endogenous protein that is part of the innate immunity and regulates wound healing. In an animal model of fibrosis, pentraxin-2 inhibits the differentiation of monocytes to fibrocytes and macrophages [59,60]. PRM-151 (Roche, Basel, Switzerland) is a recombinant human pentraxin-2 which functions as an anti-fibrotic agent in preclinical models of fibrosis and has been in clinical testing for idiopathic pulmonary fibrosis [61,62].

PRM-151 has been evaluated in MF patients in a phase 2 study. Twenty-seven patients were enrolled in the first stage of this two-stage trial and were treated with PRM-151 10 mg/kg IV monthly with or without ruxolitinib. Eighteen patients were then continued in an open label extension phase for a median of 30.9 months. The therapy was overall well tolerated. There was an average decrease in spleen size by palpation by 37% and average reduction in TSS of 54%. Nine patients had improvement in reticulin fibrosis and 8 had improvement in collagen fibrosis [63]. PRM-151 was then evaluated in a randomized, double-blind phase 2 study of 3 different dosing schedules (0.3 mg/kg, 3 mg/kg and 10 mg/kg) in patients who were previously treated or ineligible for ruxolitinib. Ninety-eight patients were randomized. The primary objective ≥1 grade improvement in bone marrow fibrosis at any time during the study occurred in 10 of 33 patients (30%) at 0.3 mg/kg, 9 of 31 pts (28%) at 3 mg/kg, and 8 of 32 pts (25%) at 10 mg/kg dose level. There was also improvement in transfusion requirements with five of 31 patients (16%) achieving red blood cell transfusion independence and six of 13 patients (46%) achieving platelet transfusion independence. Of note, the SVR_35%_ rate was not reported but 32 of 94 (34%) evaluable patients had a TSS_50%_ at any time. The most common TEAE were fatigue, cough, thrombocytopenia, and abnormal weight loss and only 51 patients (53%) completing nine cycles of therapy [64]. No future clinical trials are listed for PRM-151 in MF at the time of writing this article.

#### 3.3.2. TGFβ

Transforming growth factor β1 (TGFβ1) is secreted from megakaryocytes in MF bone marrow. Levels of TGFβ1 reach higher levels in the bone marrow from MF patients as compared with normal controls [65] and TGFβ1 promotes bone marrow fibrosis and collagen deposition in MF patients. Moreover, TGFβ can enhance dormancy in normal hematopoietic cells and inhibits megakaryocyte production, leading to a predominance of MF hematopoietic stem cells [66,67,68,69]. AVID200 (Forbius, Austin, TX, USA) is a TGFβ1 trap which binds TGFβ1 and TGFβ3, but not TGFβ2 (which is a positive regulator of hematopoiesis) [70]. In preclinical studies, AVID200 suppresses TGFβ1 signaling, leading to decreased proliferation of mesenchymal stem cells and type I collagen synthesis. In addition, AVID200 treatment led to depletion of MF *JAK2V617F* mutated mononuclear cells. In a GATA1^low^ murine model of MF, 2.5 months of AVID200 treatment led to increased total number hematopoietic and progenitor cells in the bone marrow, decreased splenic hematopoietic cells, and reduced fibrosis [71,72].

AVID200 has been explored in a phase 1 study of 12 MF patients who are resistant, intolerant, or ineligible for ruxolitinib with grade 2+ bone marrow fibrosis and platelet count > 25 × 10^9^/L. During dose escalation, no dose limiting toxicities (DLTs) occurred with 8 patients having a grade 3/4 AE which were mostly hematologic (anemia, thrombocytopenia). Spleen reduction was noted in 2 patients, which was more than 50% by palpation in both, and 5 patients had TSS_50%_. Interestingly, 8 patients had improvement in platelet count with a median increase of 48%. On four paired bone marrow specimens analyzed, there was no reduction in bone marrow fibrosis noted by central expert pathology review. An expanded efficacy analysis is being performed on two doses with results eagerly awaited [72].

Other members of the TGF-β superfamily have been targeted for different purposes in MF. TGF-β, growth differentiation factor-11 (GDF11), activin, bone morphogenetic proteins 2 (BMP2) and BMP4 also regulate erythropoiesis [73,74]. Specifically, TGF-β normally inhibits terminal erythroid differentiation by inducing apoptosis in the erythroblast [75]. Luspatercept (Acceleron Pharma, Cambridge, MA, USA) consists of the extracellular domain of the activin receptor II B fused to the Fc domain of human IgG1 and acts to trap activin and GDF11, which in turn inhibits Smad 2/3 signaling to promote differentiation of cells in the erythroid series [76,77]. Luspatercept has been evaluated in anemic MF patients in a phase 2 study. Thirty-three patients received concomitant ruxolitinib. In patients who were not transfusion dependent, the primary endpoint of a 1.5 g/dL increase hemoglobin for more than 12 weeks occurred in 2 patients (10%) not receiving ruxolitinib and 3 patients (21%) receiving ruxolitinib. In the transfusion dependent cohort, 2 patients (10%) not receiving ruxolitinib and 6 patients (32%) receiving ruxolitinib achieved transfusion independence for at least 12 weeks. Luspatercept was well tolerated, with TEAE including hypertension (11%), bone pain (8%), and diarrhea (4%) [78] observed in treated patients. A phase III, placebo-controlled trial of luspatercept in MF patients with anemia receiving ruxolitinib is currently being planned (NCT04717414).

Sotatercept (Acceleron Pharma, Cambridge, MA, USA) is a similar compound that traps ligands of activin receptor II A (instead of B, which is targeted by luspatercept). An investigator-initiated phase 2 trial of sotatercept enrolled 33 patients not receiving ruxolitinib and 21 patients in combination with ruxolitinib. Seven patients (28%) in the monotherapy cohort had a response (1.5 g/dL increase in hemoglobin for more than 12 weeks or achievement of transfusion independence if dependent at baseline). In the ruxolitinib combination cohort, there were 6 responders (32%). Similar safety findings to luspatercept were observed, with 7 patients (12.9%) having grade 3 hypertension and 2 patients (3.7%) having grade 3 myalgias [79].

#### 3.3.3. Aurora Kinase

Megakaryocyte proliferation and impaired differentiation contribute to bone marrow fibrosis in MF patients. For instance, murine models that have thrombopoietin overexpression develop fibrosis which is directly related to increase megakaryocyte mass [80] and *Gata1* deficiency leads to an accumulation of immature megakaryocytes with severe bone marrow fibrosis [81]. Aurora kinase A (AURKA) is expressed in megakaryocytes and elevated in *JAK2*, *CALR*, and *MPL* mutated cells [82,83]. Alisertib (Takeda, Tokyo, Japan) is an AURKA inhibitor that induces differentiation of megakaryocytes from PMF patients and improves bone marrow fibrosis and peripheral blood counts in a MF murine model [83].

In a phase 1, investigator-initiated study of alisertib in MF patients who are intolerant, refractory, or ineligible for a JAKi, twenty-four patients were enrolled and received a median of 7.5 cycles. The most common TEAEs were grade 3/4 neutropenia (44%), thrombocytopenia (30%), and anemia (21%), with 4% of patients experiencing vertigo, diarrhea, elevated alanine aminotransferase. In terms of response, 4 out of 14 (29%) patients with baseline splenomegaly achieved at least a 50% reduction in palpable spleen size, 2 of out 19 (11%) had an anemia response, and 7 out of 22 had a TSS_50%_. Interestingly, despite improvements in symptom and spleen burden, inflammatory cytokines were not consistently reduced and there was no correlation between cytokine reduction and response. Four out of 8 paired samples showed a decline in driver mutational burden with the other 4 showing stable VAFs. In addition, there was a reduction in bone marrow fibrosis in 5 out of 7 patients where sequential bone marrow biopsies were available [84]. Development of this agent has been halted by the sponsor but other Aurora kinase inhibitors are being explored for the treatment of MF.

### 3.4. Signaling Pathways

PI3K

Phosphoinositide 3-kinase (PI3K) is activated by tyrosine kinases on the cell membrane and in turn activates a pathway that includes Akt and mTOR [85]. This pathway regulates the cell cycle and is hyperactive in a number of cancers [86]. In MPN patients, the PI3K/Akt/mTOR pathway is constitutively active, representing a potential therapeutic target [87,88]. In addition, the combination of JAK and mTOR inhibition has synergistic anti-proliferative activity in a JAK2V617F cell line [89]. Everolimus, an mTOR inhibitor, has been evaluated in a 1/2 study of 39 patients where spleen reduction was noted in 13 patients (33.3%) with improvement in constitutional symptoms in 80% of patients [90]. However, this therapy has not been pursued further since the advent of JAKi.

Other agents that target this pathway have also been clinically tested in MF. Buparlisib, a pan-PI3K inhibitor, was evaluated in combination with ruxolitinib in the phase 1b, open-label HARMONY study. Two arms were enrolled: ruxolitinib naïve and those who have previously been treated with ruxolitinib. Sixty-three patients were enrolled in both the dose finding and expansion phase. A maximally tolerated dose (MTD) was established at ruxolitinib 15 mg twice daily and buparlisib 60 mg daily for both arms. Hematologic toxicities were common with any grade thrombocytopenia and anemia occurring in 40 patients (63.4%) and 30 patients (47.6%), respectively. Thrombocytopenia, anxiety, and depression were the primary AEs leading to study discontinuation. At the end of 24 weeks, of the patients treated at the MTD, 9 (55.6%) in the JAKi naïve arm and 3 of 7 (42.9%) in the prior JAKi arm achieved a SVR_35%_. Response rates in this trial did not suggest synergy between ruxolitinib and buparlisib, thus this therapy was not carried forward in clinical development [91].

To improve on efficacy and minimize toxicity of PI3K inhibition, parsaclisib (INCB040093, Incyte, Wilmington, DE, USA) targets only the δ subunit of PI3K, which is particularly overactive in hematologic malignancies [92]. Parsaclisib has been evaluated in a phase 2 study of 51 MF patients who had an inadequate response to ruxolitinib. Patients were randomly assigned to add parsaclisib at a dose of 10 mg or 20 mg daily for 8 weeks followed by weekly dosing or 5 mg or 20 mg daily for 8 weeks followed by 5 mg daily dosing. Median percentage decrease in spleen volume at 24 weeks was 2.5% in the daily followed by weekly dosing and 27.1% in daily dosing group. The median percentage decrease in TSS at week 12 was 14% in the daily then weekly dosing group and 51.4% in daily dosing patient. The combination was overall well tolerated. Grade 3/4 TEAEs including disseminated tuberculosis, enteritis, fatigue, hypertension, increased alanine aminotransferase, and increased aspartate aminotransferase were reported in 1 patient each in the daily followed by weekly dosing group. There was also grade 3/4 thrombocytopenia noted in 56.5% of the daily then weekly group and 11.1% of the daily group [93]. Two phase 3 studies using the daily dosing scheme are currently planned in the JAKi naïve setting (NCT04551066) and the JAKi suboptimal response setting (NCT04551053).

### 3.5. Miscellaneous Targets

#### 3.5.1. CD123

CD123 (also known as interleukin-3 receptor subunit alpha) is over-expressed in leukemic stem cells, especially in AML and blastic plasmacytoid dendritic cell neoplasm (BPDCN) [94,95]. Tagraxofusp (Elzonris, Stemline, New York, NY, USA) is a CD123-directed cytotoxin consisting of human interleukin-3 fused to truncated diphtheria toxin that was recently FDA approved for BPDCN based on the results of a clinical trial of 47 patients showing impressive response rates in both the frontline and relapsed setting [96]. CD123 is also expressed in circulating cells from patients with PMF as compared with normal control, in particular monocytes, immature myeloid cells and granulocytes [97].

Tagraxofusp has been explored in a phase 1/2 study of MF patients who were relapsed, refractory, or intolerant to JAKi. Preliminary data that was recently presented including 27 MF patients. Importantly, this therapy is available to patients with baseline thrombocytopenia, a particularly high-risk group [98]. The most common TEAEs were headache, hypoalbuminemia, increased levels of alanine aminotransferase, thrombocytopenia, and anemia. One patient experienced grade 3 capillary leak syndrome, an important AE with tagraxofusp characterized by weight gain, hypoalbuminemia, and new onset edema. Among 17 evaluable patients, 9 (53%) had reduction in spleen size and 4 had a SVR_35%_. Of 20 patients evaluable for symptom assessment, 9 (45%) had a TSS_50%_. Baseline monocytosis may be a biomarker for response, with 80% of patients with an absolute monocyte count > 1 × 10^9^/L attaining spleen reduction [99]. This clinical trial is ongoing (NCT02268253).

#### 3.5.2. Telomerase

Telomerase is a protein complex which includes human telomerase reverse transcriptase (hTERT), an RNA template, and additional specialized proteins that function to extend and maintain telomere length [100]. While inactive in normal cells, telomerase is activated in many cancer types [101]. Telomere length is globally shortened in CD34+ hematopoietic cells from patients with MPNs and telomerase activity is frequently upregulated [102]. Imetelstat (Geron, Forest City, CA, USA) is an oligonucleotide complimentary to the template region of telomerase and binds with high affinity, resulting in competitive inhibition [103]. Imetelstat has been explored in a number of solid and hematologic malignancies, albeit with lackluster clinical activity [104]. In patients with MF, however, there is compelling clinical results.

Imetelstat was first explored in a pilot study of 33 patients (16 of which had prior JAKi exposure) with intermediate-2 or high-risk disease and was administrated at 9.4 mg per kilogram either once every 3 weeks or once weekly for 4 weeks followed by once every 3 weeks for a total of 27 weeks of treatment for the core study period. Spleen size reduction greater than 50% by palpation occurred in 35% of patients. No formal symptom burden assessment was reported. Four of the 13 patients (31%) who had been dependent on red blood cell transfusions became transfusion-independent for at least 3 months. Overall, 7 patients (21%) had documented IWG-ELN response at a median time to onset of 3.5 months and a median duration of response of 18 months: 4 (12%) achieving complete remission (CR) and 3 (9%) achieving partial remission (PR) by IWG-ELN criteria. TEAE included grade 4 thrombocytopenia (18%), grade 4 neutropenia (12%), grade 3 anemia (30%), and grade 1 or 2 elevation in levels of total bilirubin (in 12%), alkaline phosphatase (in 21%), and aspartate aminotransferase (in 27%) [105]. The hepatotoxicity led to a temporary full clinical hold on imetelstat by the FDA that was later lifted.

Given these encouraging results, the multicenter, randomized phase 2 study (Imbark) was performed with two dose levels of imetelstat. Patients with relapsed/refractory MF were randomized in a single-blind fashion to either 9.4 mg/kg or 4.7 mg/kg IV every 3 weeks. At the most recent follow up, the intention-to-treat analysis of all 107 patients (n = 59 in 9.4 mg/kg arm, n = 48 in 4.7 mg/kg arm) demonstrated SVR_35%_ at 24 weeks of 0 patients in the low dose and 6 patients (10.2%) in the higher dose. TSS_50%_ occurred in 3 patients (6.3%) and 19 patients (32.2%) and reduction in bone marrow fibrosis occurred in 4 evaluable patients (20.0%) and 16 patients (43.2%) in the 4.7 mg/kg and 9.4 mg/kg arm, respectively). However, most interestingly, median OS was 28.1 months (95% CI 22.8–31.6) in the high dose group and 19.9 months (95% CI 17.1–33.9) in the low dose group. This improved survival in the 9.4 mg/kg arm significantly correlated with improved bone marrow fibrosis, and there was a trend in longer OS in patients who achieved a symptom and spleen response [106]. There appeared to be disease modifying effects of imetelstat, with reduction in driver and non-driver mutational burden and decrease in cytogenetically abnormal clones [107]. Additionally, longer OS, spleen, and symptom responses correlated with pharmacodynamic markers such as reduction in telomerase activity and hTERT expression levels [108]. This agent is being evaluated in a phase 3 controlled trial which randomizes JAKi refractory patients to either imetelstat or BAT in a 2:1 fashion with a primary outcome of OS (NCT04576156).

**Table 1:** Novel therapies in development for myelofibrosis.

Drug	Mechanism	Population	Phase	N	Clinical Efficacy *				Ongoing Clinical Trial
					SVR_35%_	TSS_50%_	BMF ≥ 1 Grade	Additional Efficacy Measures	
***Naïve to JAKi***									
CPI-0610 + ruxoloitinib	BET inhibitor	JAKi naïve	2	78	67%	57%	33%		NCT04603495
***Inadequate response to JAKi***									
Navitoclax + ruxolitinib	BCL-2/BCL-xL inhibitor	Ruxolitinib failure	2	34	27%	30%	29%		NCT04472598
CPI-0610 + ruxolitinib	BET inhibitor	Ruxolitinib sub-optimal response TI	2	26	22.2%	36.8%	-		NCT04603495
		Ruxolitinib sub-optimal response TD	2	44	20.8%	46.2%	-	21.4% TD to TI	
PRM-151 ± ruxolitinib	Recombinant Pentraxin-2	Ruxolitinib inadequate response	2	27	-	-	33.3%	Median SVR—26.1% Median TSS—64%	None
Parsaclisib + ruxolitinib	PI3Kδ inhibitor	Ruxolitinib inadequate response	2	51	-	-	-	In highest dose level: Median SVR—27.1% Median TSS—51.4%	NCT04551066
***Relapsed*, *refractory*, *or intolerant of JAKi***									
Bomedemstat	LSD-1 inhibitor	JAKi R/R	1/2	49	14%	25%	-		NCT03136185
LCL-161	SMAC mimetic	JAKi R/R	2	47	2.1%	23.4%	-		None
CPI-0610	BET inhibitor	Ruxolitinib failure TI	2	27	23.8%	47.4%	-		NCT04603495
		Ruxolitinib failure TD	2	16	0%	8.3%		34.4% TD to TI	
Tagraxofusp	CD123 directed ADC	JAKi R/R	1/2	27	24%	45%	-		NCT02268253
PRM-151	Recombinant Pentraxin-2	Ruxolitinib failure	2	98	-	-	28.1%	16% TD to TI	None
AVID-200	TGFβ trap	Ruxolitinib R/R with 2/3 BMF	1	12	-	41.7%	0%	Median maximum platelet chage 48%	NCT03895112
Imetelstat	Telomerase inhibitor	Ruxolitinib failure	2	48	10%	32.2%	43.2%		NCT04576156
Alisertib	AURKA inhibitor	Ruxolitinib failure	1	24	-	31.8%	-	29% with SVR 50% by palpation	None

* Percentages are for evaluable population. ADC = antibody-drug conjugate; BMF = bone marrow fibrosis; SVR_35%_ = spleen volume reduction of ≥35%; TD = transfusion dependent; TI = transfusion independent; TSS_50%_ = total symptom score reduction of ≥50%.

**Table 2:** International Working Group for Myelofibrosis Research and Treatment and European LeukemiaNet (IWG-ELN) Response Criteria for Myelofibrosis.

Response Categories.	Required Criteria (for All Response Categories, Benefit Must Last for ≥12 Weeks to Qualify as a Response)
**CR**	Bone marrow: * Age-adjusted normocellularity; <5% blasts; ≤grade 1 MF ^†^ and
	Peripheral blood: Hemoglobin ≥ 100 g/L and <UNL; neutrophil count ≥ 1 × 10^9^/L and <UNL;
	Platelet count ≥ 100 × 10^9^/L and <UNL; <2% immature myeloid cells ^‡^ and
	Clinical: Resolution of disease symptoms; spleen and liver not palpable; no evidence of EMH
**PR**	Peripheral blood: Hemoglobin ≥ 100 g/L and <UNL; neutrophil count ≥ 1 × 10^9^/L and <UNL; platelet count ≥ 100 × 10^9^/L and <UNL; <2% immature myeloid cells ^‡^ and
	Clinical: Resolution of disease symptoms; spleen and liver not palpable; no evidence of EMH or
	Bone marrow: * Age-adjusted normocellularity; <5% blasts; ≤grade 1 MF ^†^, and peripheral blood: Hemoglobin ≥ 85 but < 100 g/L and <UNL; neutrophil count ≥ 1 × 10^9^/L and < UNL; platelet count ≥ 50, but < 100 × 10^9^/L and <UNL; <2% immature myeloid cells ^‡^ and
	Clinical: Resolution of disease symptoms; spleen and liver not palpable; no evidence of EMH
**Clinical improvement (CI)**	The achievement of anemia, spleen or symptoms response without progressive disease or increase in severity of anemia, thrombocytopenia, or neutropenia ^§^
**Anemia response**	Transfusion-independent patients: a ≥20 g/L increase in hemoglobin level ^||^
	Transfusion-dependent patients: becoming transfusion-independent ^¶^
**Spleen response ^#^**	A baseline splenomegaly that is palpable at 5–10 cm, below the LCM, becomes not palpable ** or
	A baseline splenomegaly that is palpable at >10 cm, below the LCM, decreases by ≥50% **
	A baseline splenomegaly that is palpable at <5 cm, below the LCM, is not eligible for spleen response
	A spleen response requires confirmation by MRI or computed tomography showing ≥35% spleen volume reduction
**Symptoms response**	A ≥50% reduction in the MPN-SAF TSS ^††^
**Progressive disease ^‡‡^**	Appearance of a new splenomegaly that is palpable at least 5 cm below the LCM or
	A ≥100% increase in palpable distance, below LCM, for baseline splenomegaly of 5–10 cm or
	A 50% increase in palpable distance, below LCM, for baseline splenomegaly of >10 cm or
	Leukemic transformation confirmed by a bone marrow blast count of ≥20% or
	A peripheral blood blast content of ≥20% associated with an absolute blast count of ≥1 × 10^9^/L that lasts for at least 2 weeks
**Stable disease**	Belonging to none of the above listed response categories
**Relapse**	No longer meeting criteria for at least CI after achieving CR, PR, or CI, or
	Loss of anemia response persisting for at least 1 month or
	Loss of spleen response persisting for at least 1 month

EMH, extramedullary hematopoiesis (no evidence of EMH implies the absence of pathology- or imaging study-proven nonhepatosplenic EMH); LCM, left costal margin; UNL, upper normal limit. * Baseline and posttreatment bone marrow slides are to be interpreted at one sitting by a central review process. Cytogenetic and molecular responses are not required for CR assignment. ^†^ Grading of MF is according to the European classification Thiele et al. European consensus on grading bone marrow fibrosis and assessment of cellularity. Haematologica. 2005; 90:1128. It is underscored that the consensus definition of a CR bone marrow is to be used only in those patients in which all other criteria are met, including resolution of leukoerythroblastosis. It should also be noted that it was a particularly difficult task for the working group to reach a consensus regarding what represents a complete histologic remission. ^‡^ Immature myeloid cells constitute blasts + promyelocytes + myelocytes + metamyelocytes + nucleated red blood cells. In splenectomized patients, <5% immature myeloid cells is allowed. ^§^ See above for definitions of anemia response, spleen response, and progressive disease. Increase in severity of anemia constitutes the occurrence of new transfusion dependency or a ≥20 g/L decrease in hemoglobin level from pretreatment baseline that lasts for at least 12 weeks. Increase in severity of thrombocytopenia or neutropenia is defined as a 2-grade decline, from pretreatment baseline, in platelet count or absolute neutrophil count, according to the Common Terminology Criteria for Adverse Events (CTCAE) version 4.0. In addition, assignment to CI requires a minimum platelet count of ≥25,000 × 10^9^/L and absolute neutrophil count of ≥0.5 × 10^9^/L. ^||^ Applicable only to patients with baseline hemoglobin of <100 g/L. In patients not meeting the strict criteria for transfusion dependency at the time of study enrollment (see as follows), but have received transfusions within the previous month, the pretransfusion hemoglobin level should be used as the baseline. ^¶^ Transfusion dependency before study enrollment is defined as transfusions of at least 6 units of packed red blood cells (PRBC), in the 12 weeks prior to study enrollment, for a hemoglobin level of <85 g/L, in the absence of bleeding or treatment-induced anemia. In addition, the most recent transfusion episode must have occurred in the 28 days prior to study enrollment. Response in transfusion-dependent patients requires absence of any PRBC transfusions during any consecutive “rolling” 12-week interval during the treatment phase, capped by a hemoglobin level of ≥85 g/L. ^#^ In splenectomized patients, palpable hepatomegaly is substituted with the same measurement strategy. ** Spleen or liver responses must be confirmed by imaging studies where a ≥35% reduction in spleen volume, as assessed by MRI or CT, is required. Furthermore, a ≥35% volume reduction in the spleen or liver, by MRI or CT, constitutes a response regardless of what is reported with physical examination. ^††^ Symptoms are evaluated by the MPN-SAF TSS.17 The MPN-SAF TSS is assessed by the patients themselves and this includes fatigue, concentration, early satiety, inactivity, night sweats, itching, bone pain, abdominal discomfort, weight loss, and fevers. Scoring is from 0 (absent/as good as it can be) to 10 (worst imaginable/as bad as it can be) for each item. The MPN-SAF TSS is the summation of all the individual scores (0–100 scale). Symptoms response requires ≥50% reduction in the MPN-SAF TSS. ^‡‡^ Progressive disease assignment for splenomegaly requires confirmation my MRI or computed tomography showing a ≥25% increase in spleen volume from baseline. Baseline values for both physical examination and imaging studies refer to pretreatment baseline and not to posttreatment measurements.

## 4. Conclusions

JAKi have undoubtedly improved the lives of patients with MF. However, most patients will discontinue ruxolitinib. In the 5 year follow up from COMFORT-2, 73.3% of patients on the ruxolitinib arm had discontinued treatment primarily because of AEs (24.0%) or disease progression (21.9%) [16]. In addition, approximately 10–20% of patients with MF are ineligible for ruxolitinib or fedratinib because of thrombocytopenia and transfusion dependent anemia [109,110]. As a result, current therapeutic development in MF is focused on targets outside the JAK-STAT pathway.

In the upfront setting, efforts are underway to improve upon the symptom and spleen reductions with JAKi. Combination therapy is currently being evaluated with multiple agents including CPI-0610, parsaclisib, and navitoclax, which have ongoing phase III clinical trials in the upfront setting. Similar to multiple myeloma, rationally designed combination therapy will likely be a theme in the future treatment landscape of MF, particularly in the upfront setting.

In addition to agents previously described, there are also numerous targets with agents in clinical trials without presented data. These include the PIM inhibitors TP-3543 (NCT04176198), BH3 mimetic APG-1252 (NCT04354727), Hsp90 inhibitor PU-H71 (NCT03373877), GSK-3β inhibitor 9-ING-41, selective inhibitor of nuclear export selinexor (NCT03627403), NEDD8 inhibitor pevonedistat (NCT03386214), checkpoint inhibitor MBG453 (NCT04097821), P-selectin inhibitor crizanlizumab (NCT04097821), and TRAIL agonist ONC201, among others.

As previously discussed, patients who are relapsed, intolerant, or refractory to a JAKi have a dismal survival and higher risk of leukemic transformation [27,28,29]. In addition, splenomegaly and constitutional symptoms are less prevalent than in the JAKi naïve population [28,29,30]. Therefore, applying the same response assessment (e.g., IWG-ELN), which judges efficacy based on spleen and symptoms improvements, is not as relevant in this JAKi relapsed/refractory setting. Instead, clinical endpoints, such as OS, are more meaningful measures of clinical activity in the second-line setting. Some clinical trials in the relapsed/refractory setting are shifting the focus to survival, including the phase 3 clinical trial of imetelstat (NCT04576156). This focus away from spleen and symptom control is also encouraged by patients, as evidenced by the MPN Landmark survey where 42% of MF patient participants listed slowing the progression of their disease as the most important treatment goal, while only 7% and 6% listed symptom improvement and reduction in spleen size as their primary goal, respectively [111].

Advancing the understanding of the disease pathobiology in MF is fueling further therapeutic development in this space, including molecularly targeted therapies (e.g., *IDH2* inhibition NCT04281498). Given the importance of the JAK-STAT pathway in the disease biology of MF, it is unlikely that JAK inhibition will be abdicated as the mainstay of MF therapy. However, as demonstrated in this review, there are relevant targets outside of the JAK-STAT pathway which may be most impactful when agents are combined with a JAKi to optimize clinical response. Further work is needed to understand the efficacy, safety, and impact of novel therapies in MF. However, we are optimistic that this field, which has long been plagued by lack of available therapeutic options, is on the brink of a flourishment in therapies that can positively impact the quality and quantity of life for patients with MF.

## Data Availability

No new data were created or analyzed in this study. Data sharing is not applicable to this article.
